# Novel Disulfide Bond-Mediated Dimerization of the CARD Domain Was Revealed by the Crystal Structure of CARMA1 CARD

**DOI:** 10.1371/journal.pone.0079778

**Published:** 2013-11-05

**Authors:** Tae-ho Jang, Jin Hee Park, Hyun Ho Park

**Affiliations:** School of Biotechnology and Graduate School of Biochemistry at Yeungnam University, Gyeongsan, South Korea; University of Leeds, United Kingdom

## Abstract

CARMA1, BCL10 and MALT1 form a large molecular complex known as the CARMA1 signalosome during lymphocyte activation. Lymphocyte activation via the CARMA1 signalosome is critical to immune response and linked to many immune diseases. Despite the important role of the CARMA1 signalosome during lymphocyte activation and proliferation, limited structural information is available. Here, we report the dimeric structure of CARMA1 CARD at a resolution of 3.2 Å. Interestingly, although CARMA1 CARD has a canonical six helical-bundles structural fold similar to other CARDs, CARMA1 CARD shows the first homo-dimeric structure of CARD formed by a disulfide bond and reveals a possible biologically important homo-dimerization mechanism.

## Introduction

The CARMA1 signalosome, which is composed of CARMA1 (caspase recruitment domain (CARD)-containing MAGUK protein 1), BCL10 (B-cell lymphoma 10) and MALT1 (mucosa-associated lymphoid tissue lymphoma translocation protein 1), is a macromolecular complex responsible for lymphocyte activation [1,2]. Stimulation of lymphocyte cell surface antigen receptors (AgR) in combination with co-stimulation of co-receptors can trigger a signal for T-cell and B-cell activation through many intracellular kinases including Src, Syk, and Fyn, phosphatase, as well as many effector molecules [2-4]. Lymphocyte activation via the CARMA1 signalosome is critical to immune response, and dysregulation of the process causes many immune diseases and cancers [5,6]. It has been reported that aberrant expression of BCL10 and chromosome translocation of the MALT1 coding gene causes lymphomas of mucosa-associated lymphoid tissue [7,8]. In addition, several mutations on CARMA1 have been detected in patients with B-cell lymphoma [9]. Transcription factor NF-κB is crucial for lymphocytes activation and can be activated by the AgR-CARMA1 signalosome mediated signaling cascade [2,10]. Genetic deficiencies of NF-κB or its signaling components that act upstream of NF-κB have caused immune deficiencies, whereas over-activation of NF-κB has been linked to autoimmunity and neoplastic disorders [5,11]. NF-κB is inhibited by IκB via tight interaction in resting cells. Interaction of IκB with NF-κB causes sequestration of NF-κB in cytosol. Upon activation, IκB is phosphorylated by IκB kinase (IKK) and rapidly degraded. Removal of IκB from NF-κB leads to translocation of NF-κB from cytosol to the nucleus, where it works as a transcription factor. 

CARMA1 (also known as CARD11) is a member of the MAGUK (membrane-associated guanylate kinase) family of scaffolds that assists with recruitment and assembly of signaling molecules in the cytoplasmic membrane [12]. CARMA1 forms intracellular multi-molecular complexes with BCL10 and MALT1 that are known as CARMA1 signalosomes during lymphocytes activation and proliferation, creating a primary signaling complex for the activation of NF-κB [12]. CARMA1 contains an N-terminal CARD (CAspase Recruitment Domain) domain followed by a coiled-coil domain and a C-terminal MAGUK domain [13,14]. BCL10 is composed of an N-terminal CARD and a C-terminal Ser/Thr-rich region and has been shown to induce apoptosis and activate NF-κB [15]. MALT1 contains an N-terminal death domain (DD) followed by two immunoglobulin (IG)-like domains and a C-terminal caspase-like domain. For assembly of the CARMA1 signalosome, CARMA1 interacts directly with BCL10 via a CARD:CARD interaction and BCL10 interacts with MALT1 via an interaction between the C-terminal Ser/Thr-rich region of BCL10 and the first Ig domain of MALT1 [16,17]. CARD at CARMA1 and BCL10 and DD at MALT1 are subfamilies of the death domain (DD) superfamily, which comprises the death domain (DD) subfamily, death effector domain (DED) subfamily, caspase recruitment domain (CARD) subfamily and pyrin domain (PYD) subfamily [18-20]. The death domain superfamily is one of the largest classes of domain for protein interactions and particularly involved in the protein interactions for apoptosis and inflammation signaling pathway [21-23].

Despite the important role of the CARMA1 signalosome during lymphocytes activation and proliferation, limited structural information is available. Structural studies of the CARD-mediated assembly mechanism of the CARMA1 signalosome have been especially difficult because CARD domains are unstable under physiological conditions [24]. In this study, we report the dimeric structure of CARMA1 CARD at a resolution of 3.2 Å. Although CARMA1 CARD has a canonical six helical-bundles structural fold similar to other CARD domains, our structure shows the first homo-dimeric structure of CARD formed by a disulfide bond and reveals a possible biologically important homo-dimeric interface and novel homo-dimerization mechanism. 

## Materials and Methods

### Protein expression and purification

The expression and purification methods for CARMA1 CARD used in this study have been described in detail elsewhere [25]. Briefly, mouse CARMA1 CARD corresponding to amino acids 14-109 was amplified by PCR and inserted into the pOKD home-made vector [26]. The plasmid was then expressed in BL21 (DE3) *E. coli* competent cells by overnight induction with 0.5 mM isopropyl -D-thiogalactopyranoside (IPTG). The target protein, which contained a C-terminal His-tag, was purified by quick two step chromatography, nickel affinity and gel-filtration chromatography using a Superdex200 gel filtration column (GE Healthcare) that had been pre-equilibrated with a solution of 20 mM Tris at pH 8.0 and 150 mM NaCl. The eluted target protein was collected and concentrated to 5 mg ml^-1^. 

### Crystallization and data collection

The crystallization conditions were initially screened at 20°C by the hanging drop vapor-diffusion method using various screening kits. Initial crystals were grown on the plates by equilibrating a mixture containing 1 μl of protein solution (5-6 mg ml^-1^ protein in 20 mM Tris at pH 8.0, 150 mM NaCl) and 1 μl of a reservoir solution containing 0.2 M ammonium sulfate, 0.1 M MES at pH 6.5, and 30% polyethylene glycol (PEG) monomethyl ether (MME) 5,000 against 0.4 ml of reservoir solution. Crystallization was further optimized by searching over a range of concentrations of protein, PEG MME 5,000, and ammonium sulfate. Selenomethionine-substituted CARMA1 CARD was produced using a previously established method [27] and crystallized similarly. A single-wavelength anomalous diffraction (SAD) data set was collected at the BL-4A beamline at Pohang Accelerator Laboratory (PAL), Republic of Korea. Data processing and scaling were carried out using the HKL2000 package [28]. A 3.5 Å native data set was also collected at PAL. The details of crystallization and data collection were introduced previously [25]. 

### Structure determination and analysis

Selenium positions were found with HKL2MAP [29] using the dataset collected at the peak wavelength. Phase calculation and phase improvement were performed using the SOLVE and RESOLVE programs [30]. Approximately 70% of the structure was auto-traced. Model building and refinement were performed in COOT [31] and Refmac5 [32], respectively. Water molecules were added automatically with the ARP/wARP function in Refmac5 and subsequently examined manually for reasonable hydrogen bonding possibilities. Geometry was checked by PROCHECK and used to guide the refinement procedure. Refinement statistics are listed in Table 1. Ribbon diagrams and molecular surface representations were generated using the Pymol Molecular Graphics System (2002; DeLano Scientific, San Carlos, USA).

**Table 1:** Crystallographic statistics.

**Data collection**	Se-Met	Native
Space group	*P2_1_2_1_2_1_*	*P2_1_2_1_2_1_*
Cell dimensions		
*a*, *b*, *c*	45.8Å, 53.9Å, 92.8Å	45.7Å, 53.4Å, 91.9Å
Resolution	50-3.2 Å	50-3.5Å
*†* *R*_sym_	15.9 % (55 %)	11.0 % (40.6 %)
*†I*/σ*I*	21.0 (5.0)	28.3 (7.1)
*†*Completeness	99.9 % (100 %)	99.9 % (100 %)
*†*Redundancy	6.8 (6.9)	8.9 (8.8)
**Refinement**		
Resolution	50-3.2Å	
No. reflections used (completeness)	4116 (99.7%)	
*R*_work_/*R*_free_	29.1%/32.4%	
No. atoms		
Protein	1334	
Water	14	
Average B-factors		
Protein	49.6 Å^2^	
Water and other small molecules	22.3Å^2^	
R.M.S. deviations		
Bond lengths	0.015Å	
Bond angles	1.632°	
Ramachandran Plot		
Most favored regions	96 %	
Additional allowed regions	4 %	

† Highest resolution shell is shown in parenthesis.

### Sequence alignment

The amino acid sequence of CARDs was analyzed using Clustal W (http://www.ebi.ac.kr/Tools/clustalw2/index.html).

### Disulfide bond assay using gel-filtration chromatography

For gel filtration analysis to detect disulfide bond-mediated dimerization, purified CARMA1 CARD was applied to a gel-filtration column (Superdex 200 HR 10/30, GE Healthcare) that had been pre-equilibrated under two different conditions, one with 20 mM Tris-HCl 8.0 and 150 mM NaCl and another with 20 mM Tris-HCl 8.0, 150 mM NaCl and 5 mM DTT. 

### Native PAGE shift assay

Disulfide bond-mediated dimerization of CARMA1 CARD was monitored by native (non-denaturing) PAGE on a PhastSystem (GE Healthcare) with pre-made 8–25% acrylamide gradient gels (GE Healthcare). Coomassie Brilliant Blue was used for staining and to detect dimer and monomer bands. 

### MALS

The molar mass of CARMA1 CARD in the presence or absence of DTT was determined by MALS. Briefly, highly purified CARMA1 CARD was injected onto a Superdex 200 HR 10/30 gel filtration column (GE healthcare) that had been equilibrated with buffer containing 20 mM Tris HCl and 150 mM NaCl with or without DTT. The chromatography system was coupled to a MALS detector (mini-DAWN EOS) and a refractive index detector (Optilab DSP) (Wyatt Technology). 

### Protein data bank accession code

Coordinate and structural factor have been deposited in the RCSB Protein Data Bank under accession code 4JUP. 

## Results and Discussion

### CARMA1 CARD structure

**Figure 1:**
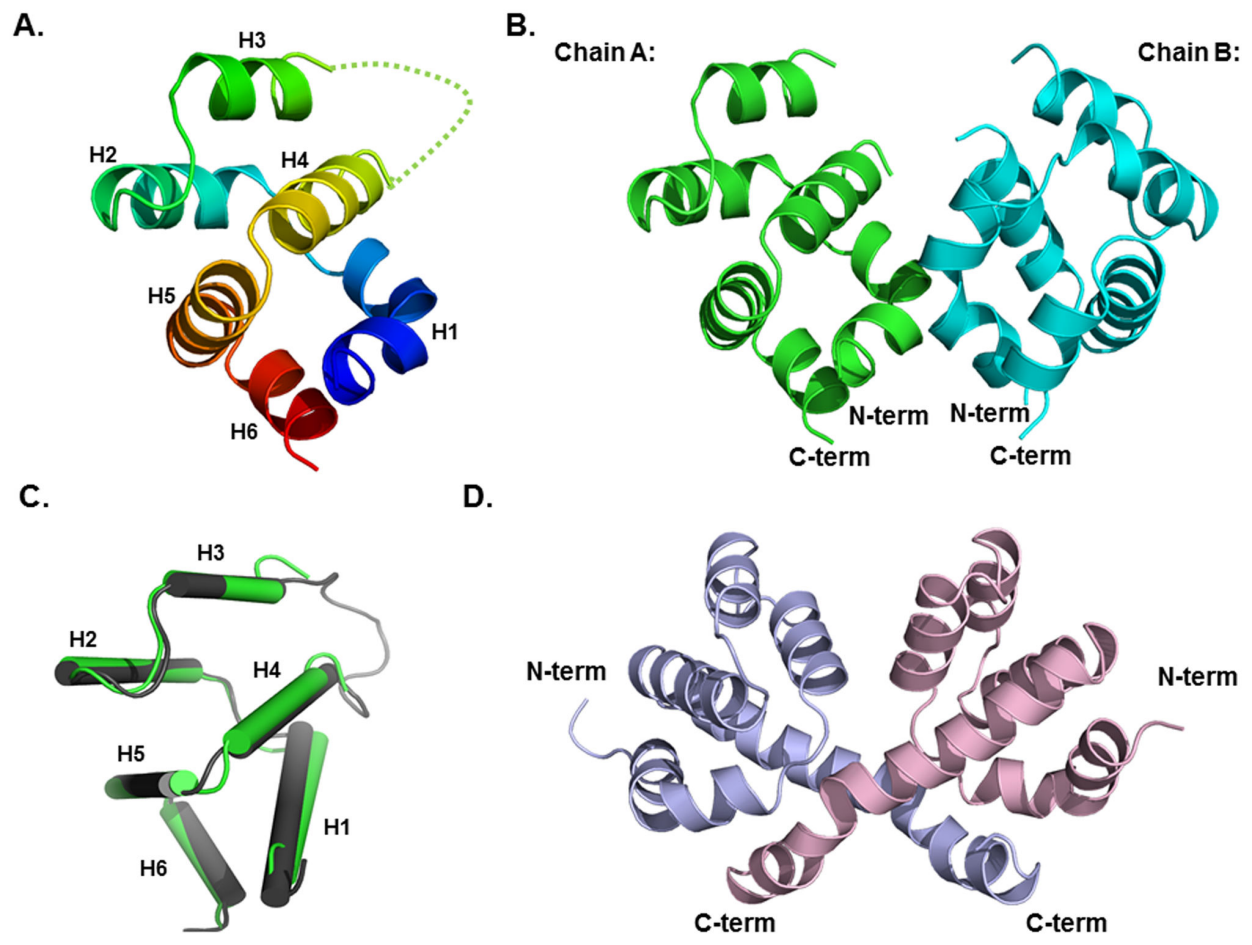
Crystal structure of CARMA1 CARD. A. Ribbon diagram of CARMA1 CARD. The chain from N- to C-termini is colored by the spectrum from blue to red. Helices from H1 to H6 are labeled. Missing residues are shown as a green dotted-line B. Dimer structure of CARMA1 CARD. Chain A (green color) and Chain B (cyan color) are shown separately. C. Structural comparison between monomeric CARMA1 CARD (colored in gray) and dimeric CARMA1 CARD (colored in green). D. Structure of NOD1 CARD.

The 3.2 Å crystal structure of CARMA1 CARD was solved using a single-wavelength anomalous diffraction (SAD) method and refined to an *R*_work_ of 29.1% and *R*_free_ of 32.4%. The high resolution structure of CARMA1 CARD showed that it forms the canonical six-helical bundle fold comprising six helices, H1 to H6, which is characteristic of the DD superfamily (Figure 1A and 1B). There was one dimer in the asymmetric unit, referred to as chain A and chain B (Figure 1B). Model chains were built from residue 22 to residue 108. A flexible loop formed by residue 64 to residue 70 was missing from our model. The electron density of that region was too weak to trace, indicating that the loop is extraordinarily flexible relative to those of other CARD domains (Figure 1A and 1B). Chain A and Chain B formed a symmetric dimer. H3 and H6 were shorter than other helices. The N and C termini of CARMA1 CARD were located on the same side of the molecule. The six helices comprising residues 24-35, 39-49, 54-61, 73-83, 85-98, and 100-107 were numbered H1, H2, H3, H4, H5, and H6 (Figure 1A). Helix bundles tightly packed by a central hydrophobic core were formed by several conserved residues including V26, L33, I37, T43, I52, L76, L77, F92, L93, L95, and Y103 . This hydrophobic core supports the location of each helix and stabilizes the conformation of CARMA1 CARD. Three loops, including residues 36-38 (H1-H2 loop), 50-53 (H2-H3 loop), 62-72 (H3-H4 loop), and two linkers, including 84-85 (H4-H5 loop) and 99-100 (H5-H6 loop), connect the six helices. The missing H3-H4 loop was the longest loop. Interestingly, H1 of CARMA 1 CARD was bent in the middle. A bent H1 was previously detected in the structure of other CARDs including Apaf-1 CARD [33] and NOD1 CARD [34], but never in other death domain superfamily members, including DD, PYD and DED. These findings indicate that the bent H1 might be a distinct feature of the CARD subfamily. Recently, monomeric CARMA1 CARD structure was elucidated [35]. The RMSD value of superimposed structure between monomeric CARMA1 CARD and dimeric CARMA1 CARD was 0.7 Å indicating that the overall structure was almost identical (Figure 1C). The dimeric CARD was previously reported in the structure of NOD1 CARD [36]. In this structure, unusually extended sixth helix of NOD1 CARD is swapped between two monomers (Figure 1D). 

### Disulfide bond-mediated dimerization of CARMA1 CARD

**Figure 2:**
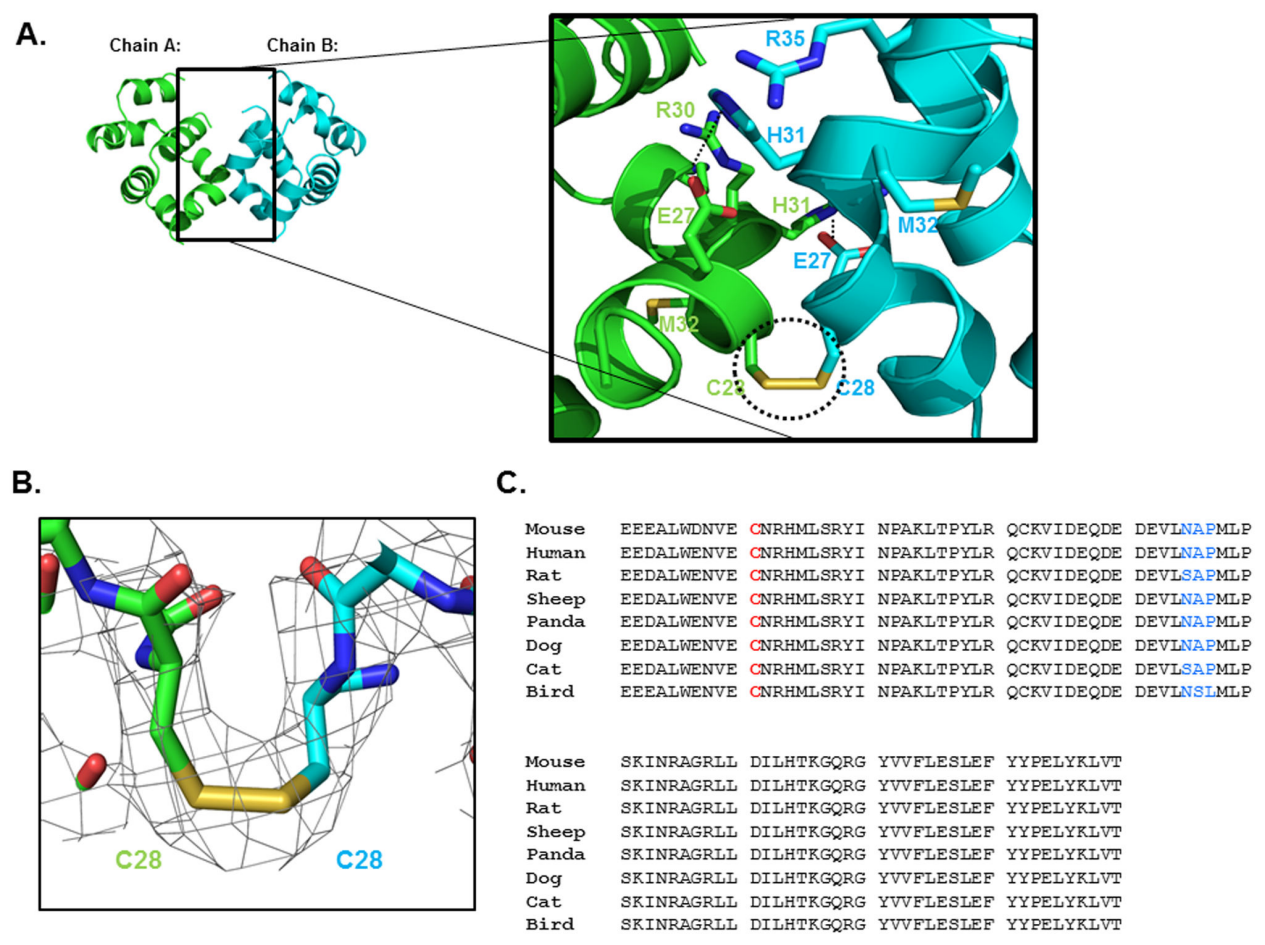
Dimeric interface of the structure of CARMA1 CARD. A. The dimeric structure of CARMA1 CARD and close-up view of the interacting residues in the interface between dimers (cyan for chain B and green for chain A). The residues involved in the contact are shown as cyan (from chain B) and green (from chain A) sticks. Hydrogen bonds formed between E27 from one chain and H31 from counterpart are shown as dashed lines. The disulfide bond formed by C28 from chain A and C28 from chain B is shown as a black dashed-circle. B. Close-up view of the disulfide bond between C28 (chain A) and C28 (chain B). The electron density map is shown as a gray color mesh. C. Conserved cysteine residue involved in the disulfide bond. The conserved cysteine residue cross species is highlighted by red color. Perfectly conserved residues are shown in black and residues that are not perfectly conserved are blue.

The crystal structure of CARMA1 CARD revealed interesting information pertaining to the novel homo-dimeric interfaces. The two CARMA1 CARD structures in the asymmetric unit form a symmetric dimer. Not many interaction forces were detected at the interaction interfaces. An electrostatic interaction between H31 from one molecule and E27 from another molecule was the only non-covalent bond detected at the interface (Figure 2A). A total dimer surface buries 698 Å^2^ (a monomer surface area of 349 Å^2^), which represents 7.5% of the dimer surface area calculated by PDBePISA [37]. Interestingly, an unexpected disulfide-bond formed by C28 from each molecule was detected (Figure 2B). The disulfide bond observed in the structure was formed between helix1. Although stable dimeric forms of the death domain superfamily have been reported [38], this is the first report of disulfide bond-mediated homo-dimerization. C28, which participated in the formation of the disulfide bond, was conserved across species, indicating that this disulfide bond might be important to the function of CARMA1 (Figure 2C). Reactive oxygen species (ROS) were recently shown to activate formation of the molecular complex, and this activation is mediated by the death domain superfamily, including the CARD domain. Inflammasome mediated inflammation activation by ROS is the representative case [39]. Two death domain superfamily members, PYD and CARD, are involved in the protein-protein interaction leading to the inflammasome formation. It has been proposed that ROS directly controls the oligomeric state of NALP3 PYD and regulates formation of the inflammasome. Based on the results of a previous study, it is possible that formation of the CARMA1 signalosome is controlled by ROS, which can regulate the oligomeric states of CARMA1. However, further cellular studies have to be conducted to confirm this. 

**Figure 3:**
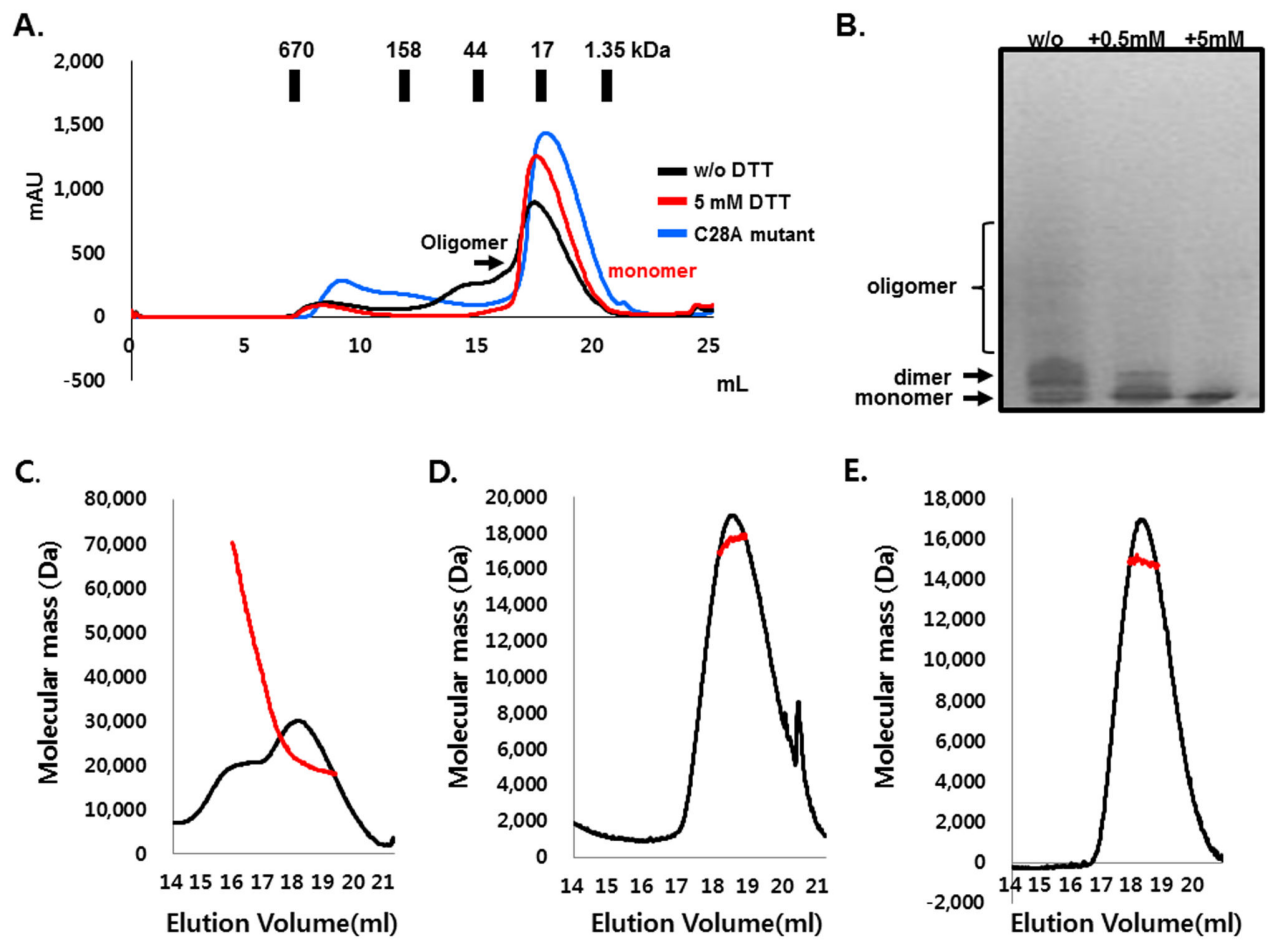
Formation of dimeric CARMA1 CARD in vitro. A. Gel-filtration chromatography showed that CARMA1 CARD forms a dimeric and further oligoemric states without reducing agent (black line). The dimeric form of CARMA1 CARD became monometic in response to the addition of reducing agent (red line). C28A mutant also became monomeric in solution. B. Native-PAGE confirmed that CARMA1 CARD exists as dimer in solution and became a monomer by adding DTT. The position of the dimer form and monomeric form on native-PAGE are indicated. The amounts of DTT added are shown above the picture. w/o: without DTT. +: with DTT. C. Determination of the molecular mass of the CARMA1 CARD in the absence of reducing agent by multi-angle light scattering. D. Determination of the molecular mass of the CARMA1 CARD in the presence of reducing agent (5 mM DTT) by multi-angle light scattering. E. Determination of the molecular mass of C28A mutant by multi-angle light scattering.

Disulfide bond-mediated oligomerization was also detected in solution. Despite its complexity, dimeric (even highly oligomeric) and monomeric CARMA1 CARD co-exists in solution without reducing agent dithiothreitol (DTT), which breaks the disulfide bond. However, gel-filtration chromatography revealed that the addition of 5 mM DTT resulted in CARMA1 CARD becoming monomeric (Figure 3A). C28A mutant also was not able to produce oligomeric peaks on the profile of gel-filtration chromatography. The reducing agent mediated monomeric change of CARMA1 CARD was also detected upon native-PAGE analysis. Specifically, dimeric CARMA1 CARD became the monomeric form in a DTT concentration dependent manner (Figure 3B). To confirm the previous results, we calculated the absolute molecular weight of CARMA1 CARD with or without reducing agent using multi-angle light scattering (MALS). C28A mutant was also measured with MALS. The calculated monomeric molecular weight of CARMA1 CARD including the C-terminal His-tag was 12.35 kDa, while that of MALS was 22.18 kDa (0.7% fitting error) without DTT and 17.12 kDa with 5 mM DTT (Figure 3C and 3D). C28A was 14.21 kDa (Figure 3E). As shown in Figure 3C, CARMA1 CARD exists as dimerand further oligomer mixture in solution and was all changed to the monomeric form by the addition of DTT and by mutating C28 to Alanine, indicating that CARMA1 CARD forms a stable dimer in solution and that the dimerization is mediated by a disulfide bond. 

### Comparison with other CARD structures

**Figure 4:**
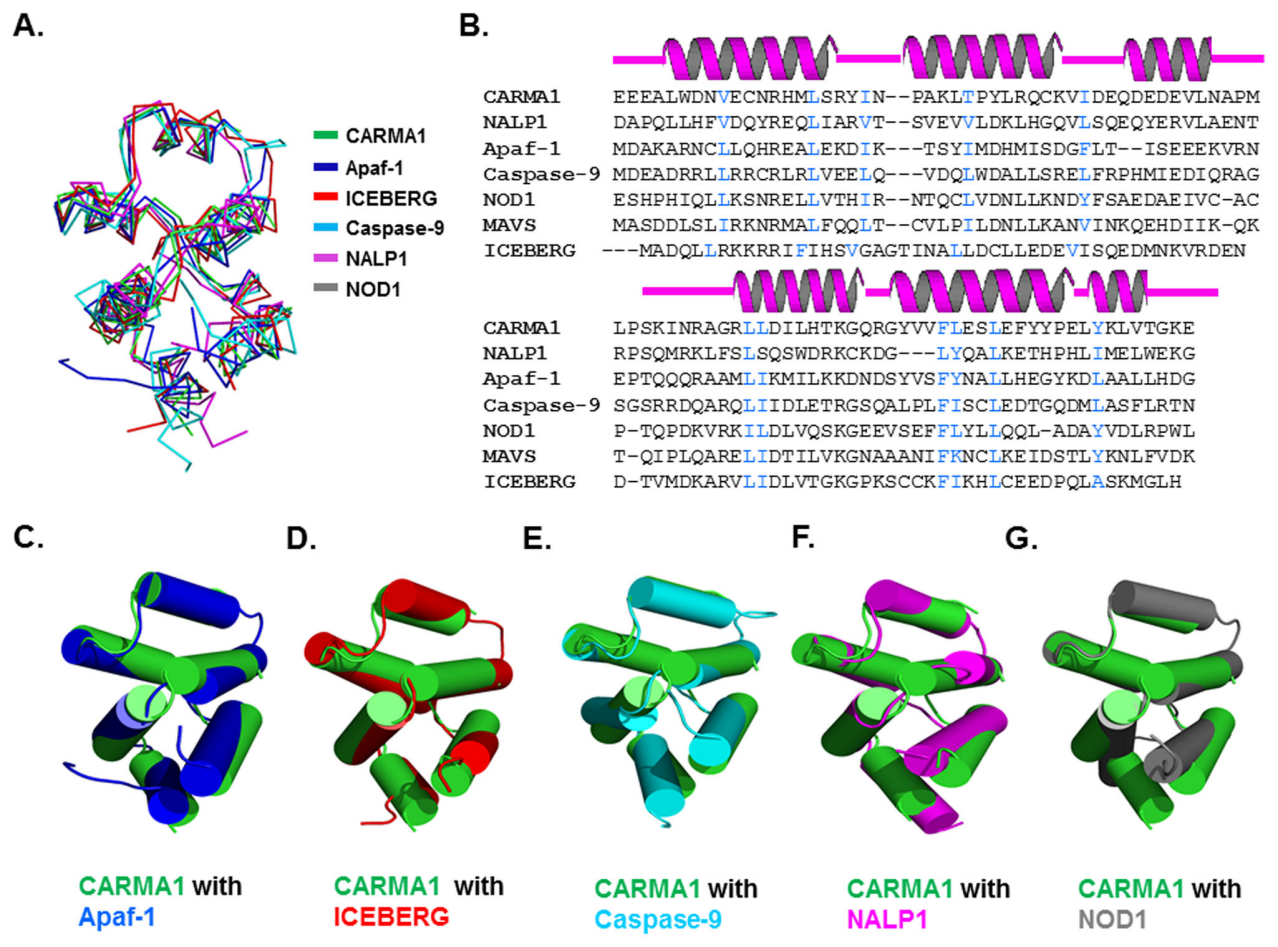
Superposition of CARMA1 CARD with their structural homologues. A. CARMA1 CARD (green color) and five structural homologues are superimposed. B. Structural-based sequence alignment. Structure-based sequence alignment of CARMA1 CARD with other CARD domains. Secondary structures (helices H1 to H6) are shown above the sequences. Residues at the hydrophobic core are shown in blue. C-G. Pair-wise structural comparisons were also performed. CARMA1 CARD is green and each counterpart is blue for Apaf-1 CARD (B), red for ICEBERG (C), cyan for caspase-9 CARD (D), magenta for NALP1 CARD (E), and gray for NOD1 CARD (F).

A structural homology search using the DALI server [40] revealed that the structure of CARMA1 CARD is highly similar to that of other CARDs, although the sequence identity is low (less than 25 % identity). The top ten selected matches, with high Z-scores, were as follows: APAF-1, ICEBERG, Caspase-9, NOD1, CED4, RIG1, CED9, NALP1, c-IAP1, and MAVS (Table 2). UNC5H2 DD was the most structurally similar protein outside of members of the CARD family. All CARDs complosed of six helixes bundle (Figure 4A). Residues buried within the hydrophobic core are conserved among CARDs (Figure 4B)Pair-wise structural alignments between CARMA1 CARD and these other representative CARDs revealed that the helices and loops in CARMA1 CARD slightly differed in length and orientation (Figure 4). For example, when compared with APAF-1 and Caspase-9, helix H6 of CARMA1 CARD showed differences in orientation, as did H5 from Caspase-9. Differences in length were also detected at H6 of ICEBERG and H4 of NALP1. The greatest differences were detected at the H3-H4 loop (Figure 4). Specifically, the length of the H3-H4 loop of CARMA1 CARD was similar to that of Caspase-9, possessing a relatively long loop compared to those of APAF-1, ICEBERG, NALP1, and NOD1. In a previous study, the structure of NOD1 CARD showed a missing H6 that was combined with H5 [34]. This merging of H5-H6 was not detected in the present study, indicating that H5-H6 merging is unusual and might be an artifact. 

**Table 2:** Structural similarity search using DALI [39].

Proteins and accession numbers	Z-score	RMSD (Å)	Identity (%)	References
APAF-1 CARD (3ygs-c)	13.0	1.4	22	[41]
ICEBERG (1dgn)	11.3	1.8	20	[43]
Caspase-9 CARD (3ygs-p)	11.1	1.7	18	[41]
NOD1 CARD (4e9m)	11.0	1.4	23	[34]
CED4 CARD (3lqq)	10.9	1.7	12	[44]
RIG1 CARD (4a2q)	10.8	1.7	14	[45]
CED9 CARD (2a5y)	10.6	1.7	12	[46]
NALP1 CARD (3kat)	10.5	2.0	14	[47]
c-IAP1 CARD (3t6p)	10.1	1.6	24	[48]
MAVS CARD (2vgq)	9.4	1.9	15	[49]
UNC5H2 DD (1wmg)	8.1	2.4	14	[50]

### Model of the interaction between CARMA1 CARD and BCL10 CARD for assembly of the CARMA1 signalosome

**Figure 5:**
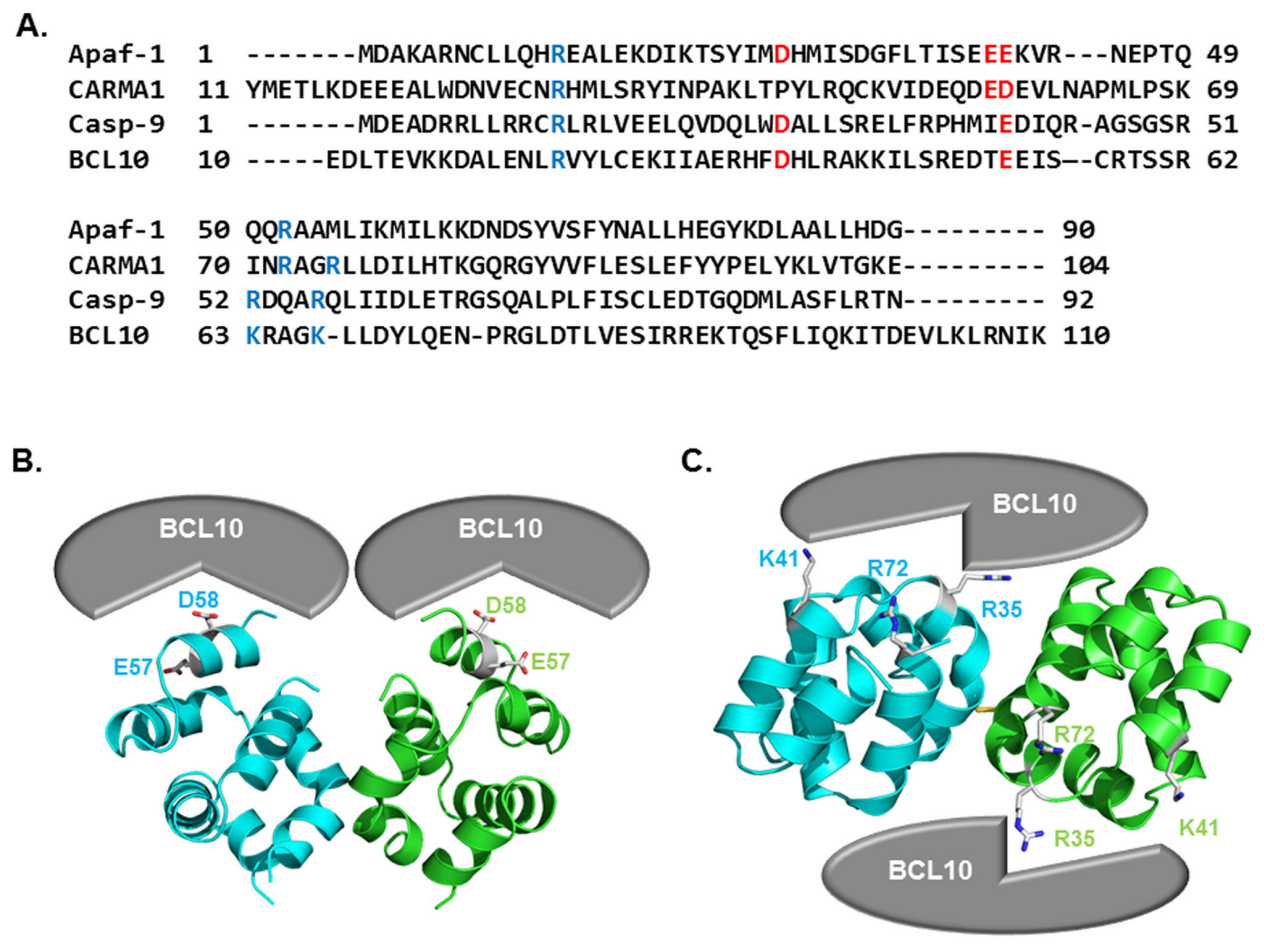
Model of the interaction between CARMA1 CARD and BCL10 CARD. A. Sequence alignment between CARDs. B and C. Model of interaction between CARMA1 CARD and BCL10 CARD. Two different models are introduced. The acidic patch of CARMA1 CARD can be used to interact with the basic patch of BCL10 CARD (B). The basic patch of CARMA1 CARD can also be involved in the interaction with BCL10 CARD (C).

Because the CARD domain-mediated interaction between CARMA1 CARD and BCL10 CARD is important to understand the oligomerization of the CARMA1 signalosome for immune cell signaling, the structural information describing CARMA1 CARD:BCL10 CARD complex is critical. Without the current available complex structure, we estimated the interaction between CARMA1 CARD and BCL10 CARD based on the dimeric current structure of CARMA1 CARD. To determine possible interactions between CARMA1 CARD and BCL10 CARD, the previously solved structure of the Apaf-1 CARD:Caspase-9 CARD complex, which is the only available structure of the CARD complex, was used. In the Apaf-1 CARD:Caspase-9 CARD complex, the interaction is largely mediated by charge complementarity [41]. Three positively charged residues in caspase-9 CARD, R13, R52, and R56, and two negatively charged residues, D27 and E40, in Apaf-1 CARD are crucial for the interaction. All charged residues that are critical for the interaction between Apaf-1 CARD and caspase-9 CARD are conserved at CARMA1 CARD (E56, D58, R20, R72, and R75) and BCL10 CARD (D38, E52, R24, K63, and K67) based on the sequence alignment (Figure 5A). The gross features of the electrostatic surface of CARMA1 CARD, BCL10 CARD, Apaf-1 CARD and Caspase-9 CARD are very similar. If dimeric CARMA1 CARD uses the acidic patch (E56) for interaction with the basic patch of BCL10 (R24, K63 and K67), the interaction mode looks like the model introduced at Figure 5B. However, if dimeric CARMA1 CARD uses the basic patch formed by R20, R72 and R75 to interact with the acidic patch of BCL10 CARD (D38 and E52), the interaction mode looks like the model introduced at Figure 5C. 

Based on the dimeric structure of CARMA1 CARD and follow-up study, we suggest the molecular mechanism of the assembly of the CARMA1 signalosome, which is the critical signaling molecular complex in the immune system. To obtain clearer insight into formation of the CARMA1 signalosome, further efforts to solve the complex structure are needed. Finally, previous study showed that oligomerization of CARMA1 is mediated by coiled-coil domain of CARMA1 [42]. Based on the current structural study, dimerization of CARMA1 CARD by disulfide-bond also can help CARMA1 oligomerization and further formation of CARMA1 signalosome. 
